# Nurse’s scope of practice in home-based care: a qualitative descriptive design – the DECIDE project

**DOI:** 10.1186/s12912-026-04590-3

**Published:** 2026-03-28

**Authors:** Ida Røed Flyum, Veronica Pavedahl, Gunilla Borglin, Edith Roth Gjevjon, Anna Josse Eklund

**Affiliations:** 1 Department of Undergraduate Studies, Lovisenberg Diaconal University College, Lovisenberggata 15B, Oslo, NO-0456 Norway; 2 Department of Nursing, Faculty of Health, Nature and Technical Science, Institute of Health Sciences, Karlstad University, Universitetsgatan 2, Karlstad, 651 88 Sweden

**Keywords:** Activities, Clinical decision-making, Continuity of care, Dyadic analysis, Framework analysis, Non-registered nursing staff, Nursing process, Qualitative research, Registered nurses, Role

## Abstract

**Background:**

Nurses’ understanding of their scope of practice is essential for coordinating safe and optimal home-based care for older people with complex needs. Contextual modulators, workload pressures, and limited opportunities for clinical leadership may hinder enactment of core aspects of this scope. Uncertainty regarding nurses’ scope of practice persists, particularly in home-based care. The aim of this study was to explore how nurses experience their own and each other’s scopes of practice, and what characterises their scope of practice in home-based care.

**Methods:**

A qualitative descriptive design using focused individual interviews was employed with eight registered nurses and eight non-registered nurses from three home-based nursing teams in south-east Norway. Although interviews were conducted individually, data were analysed dyadically using an adapted Framework Method to enable systematic comparison of perspectives.

**Results:**

Nurses’ scope of practice was characterised by ongoing clinical observation, assessment, and response to change, supported by continuity of visits and in-depth knowledge of service users. Activities ranged from household chores and basic care to advanced clinical procedures, with substantial overlap between the nurses. Registered nurses articulated greater analytic depth and system-level accountability. Despite shared conceptual understandings, nurses experienced a parallel scope of practice with divergent responsibilities. Registered nurses assumed primary accountability in acute, complex, and transitional care situations, and tensions were evident regarding responsibility distribution and decision-making.

**Conclusions:**

Scope of practice in home-based care was dynamic and context-dependent, enacted through an embodied nursing process reflecting rapid, experience-based cognition and analytic decision-making. Although nurses described shared understandings, enactment involved overlapping activities alongside distinct responsibilities. Registered nurses’ scope of practice extended beyond direct care to coordination, clinical leadership, and accountability for care completion. These findings highlight risks of implicit responsibility shifting and emphasise the need to clearly define and empirically ground nurses’ scope of practice to support safe practice, leadership, workforce sustainability, and future research on continuity, responsibility, and decision-making in complex care settings.

**Trial registration:**

Not applicable.

## Background

Nurses’ internalised understanding of their Scope of Practice (SoP) in home-based care is fundamental to their ability to coordinate and deliver meaningful care for the growing number of older people with complex health needs. In this study, SoP is conceptualised as nurses’ responsibilities and authority (role), the activities and tasks they perform (function), and their capacity for clinical decision-making [1–3]. Internalising these components is particularly important in the inherently multiprofessional context of home-based care, where registered nurses (RNs), who are anticipated to take primary clinical leadership, need to collaborate closely with various occupational groups, most importantly healthcare workers, here referred to as non-Registered Nurses (non-RNs, i.e., vocational training without the RNs credential). In the following, when both registered and non-registered personnel are relevant, the term “nurse(s)” will be used to refer collectively to these roles. Research across home- and facility-based care indicates that RNs frequently adapt and extend their roles to meet complex care needs and organisational demands, performing additional activities and decision-making beyond formal task lists, which is associated with higher workload and may impact care quality [4, 5]. RNs must advocate for their professional remit and clarify role boundaries to prevent confusion [6] and avoid the risk of nursing roles being diluted or reduced to merely “filling gaps” in care [7, 8], potentially diminishing the recognition of nursing as a distinct professional discipline. Non-RNs are often described as a valuable extension of RNs [9], primarily providing and monitoring direct care and reporting to RNs, highlighting the importance of understanding both similarities and differences in their respective SoP. Such understanding provides RNs with a foundation for fulfilling their responsibilities for clinical leadership and decision-making. At the organisational level, this also highlights the importance of clear task allocation, supportive leadership, and structured delegation practices to ensure that RNs and non-RNs can work effectively within their respective scopes of practice, maintain care quality, and reduce the risk of role ambiguity or work overload.

Clinical decision-making and clinical leadership are core components of RNs’ scope of practice and, according to the literature, key elements that distinguish them from non-RN colleagues. Studies in both home- and facility-based care show that RNs’ ability to enact their core responsibilities can be constrained by modulators such as stress, fatigue, and limited opportunities for clinical leadership [4, 5] Flyum et al., manuscript under review. Despite differences in care contexts, these studies report similar challenges in managing older people with complex care needs, highlighting the relevance of these findings across settings. RNs appear to spend a disproportionate amount of time on assessment while devoting minimal time to evaluating care. This is important, as effective decision-making and clinical leadership are autonomous RN competencies central to care quality and outcomes. Clinical decision-making is a complex, multidimensional process that underpins all nursing care, guiding decisions about what to do, when, and how [10, 11], as well as directing clinical leadership and delegation to non-RNs. Nurses in home- and facility-based care have been found to characterise their practice as reactive, intuitive, and experience-based, with limited articulation of the rationale underlying their clinical decision-making [12]. Reliance on this approach without the integration of more analytic processes may increase the risk of suboptimal decisions, particularly in high-stress situations or when nurses lack the necessary experience and competence.

With the rising complexity of home-based care—driven by an increasing number of older people with chronic conditions and growing resource constraints—RNs’ competence and confidence in their SoP are increasingly important. Insufficient internalisation of their professional role may contribute to rationed care [13] and unmet care needs [14], with potential consequences for both care recipients and nurses, regardless of role or educational background.

Contemporary RNs continue to grapple with foundational questions regarding what constitutes their work, why it is performed, and how it should be enacted. Although efforts to define and operationalise nursing practice have a long history [e.g., 15, 2, 3], scholars argue that the nursing profession still struggles to articulate who they are as professionals, why they do what they do [16], and the essential nature of their practice—the “what” of nursing [17]. Some scholars have described nursing work as ‘invisible’ [18], which may obscure the responsibilities, decision-making authority, and professional recognition that are central to SoP.

To frame the nurses’ practice, we drew on two complementary references. Malone and Cliffe’s Framework for Remote and Isolated Practice [1] provides guidance for understanding professional roles in contexts with limited resources, emphasising accountability and collaboration. The Royal College of Nursing’s updated definition of nursing [2] clarifies RNs’ remit, focusing on role, responsibilities, and clinical decision-making. Together, these references offer a conceptual lens that allows the operationalisation of SoP in terms of role (responsibilities, authorisation) and function (e.g., activities, tasks), while avoiding a prescriptive or rigid framework [19, 20]. This lens guided exploration of the nurses’ own interpretations of their practice in home-based care. However, it remains unclear how issues related to role boundaries, responsibility distribution, and decision-making manifest in everyday practice for nurses in home-based care, highlighting the need for detailed empirical examination [21]. The aim of this study was to explore how nurses experience their own and each other’s scopes of practice, and what characterises their scope of practice in home-based care.

## Methods

### Study design

A qualitative descriptive design was used to capture practice-proximate data reflecting nurses’ experiences in home-based care [22]. Data were collected through individual focused interviews [23]. Although interviews were conducted individually, the data were subsequently analysed using the Framework Method for non-dyadic data [24], with dyads constructed *a posteriori* to allow systematic comparison of nurses’ perspectives [see Data Analysis section]. SoP was used as a guiding concept to inform data collection and analysis, operationalised as role (responsibilities and authorisations), function (tasks, activities), and decision-making, without applying a formal theoretical framework [19, 20]. The study is reported in accordance with the Standards for Reporting Qualitative Research (SRQR) [25].

### Study setting and recruitment strategies

This study was conducted in three Norwegian home-based nursing teams within a municipal home healthcare service. In Norway, home-based nursing care predominantly serves older people, many of whom have complex care needs [26]. Primary healthcare is organised at the municipal level, with municipalities legally responsible for the provision of home-based nursing care. Services are publicly funded and delivered through multidisciplinary teams. In this context, care is primarily provided by Registered Nurses (RNs), who hold a three-year bachelor’s degree, and healthcare workers with vocational training (non-RNs), who complete two years of upper-secondary education followed by an apprenticeship leading to a trade certificate and licensure [27, 28]. RNs hold formal responsibility for clinical assessment, care planning, and clinical decision-making, while non-RNs provide much of the direct daily care. The formal distinction between them is anchored in national health legislation. The Health Personnel Act assigns RNs independent responsibility for professional judgement, clinical decision-making, and delegation of care, whereas non-RNs practise under delegation and supervision [29, 30]. This legal framework is further clarified through national guidance and regulatory oversight provided by the Norwegian Directorate of Health and the Norwegian Board of Health Supervision [31, 32]. In the home-based context, nurses often work autonomously in patients’ homes, frequently without immediate physician presence, which places substantial demands on clinical decision-making, coordination, and role clarification. This organisational and regulatory context shapes how scope of practice is enacted in everyday home-based care and forms the backdrop for the experiences explored in this study.

### Participants

Participants were purposively recruited [33, 34] from a pool of thirty-two nurses who had previously taken part in a larger two-step research project – the DECIDE project. The first step [Flyum et al., manuscript under review] involved direct structured observations focusing, amongst others, on the same overarching nursing phenomena as explored in this study, whereas the current study represents the second step. All 16 nurses included here had consequently been observed in their work during the first step of the DECIDE project. The insights from these direct structured observations provided contextual knowledge informing the study design, interview question, and probing prompts. Following this contextual preparation, eight nurse dyads were constructed *a posteriori* by randomly pairing one RN and one non-RN from the same nursing team and who had been observed on the same day. Alternative pairings were possible, but recruitment was stopped once 16 participants were included. This sample size was considered sufficient based on information power, as previous research suggests that meaningful themes can be captured after 6–12 interviews when participants are relatively homogeneous and the study aim is specific [35, 36].

Inclusion criteria were: (1) permanent or long-term temporary employment within one of the three participating nursing teams, (2) direct involvement in nursing care for older people with complex needs, and (3) proficiency in Norwegian. Recruitment was carried out in collaboration with team leaders and professional development RNs and included informational meetings and distribution of flyers in team break rooms.

### Data collection

Data were collected through individual focused interviews [23] conducted separately, resulting in non-dyadic data [24]. Interviews were conducted between March and June 2025 and lasted between 43 and 112 min (mean = 75 min). Time and location were arranged collaboratively with each participant. All interviews were audio-recorded and transcribed using Intelligent Speech Recognition Technology (ISRT) [37], followed by manual verification to ensure accuracy. Interviews were conducted in Norwegian, and the team verified translations into English for analysis to ensure accuracy.

The first author conducted all interviews and had previously carried out the direct structured observations in Step I; coding and analysis were undertaken collaboratively by the research team, with the last author overseeing the interpretive process. Although observations in Step I informed the interview focus, potential influence on interpretation was addressed through collaborative analysis and ongoing reflexive discussions within the research team. To ensure data quality, two senior researchers reviewed the first two interview recordings; both were assessed as meeting quality standards and were retained in the dataset.

The interviews were guided by a single open-ended question: I am interested in hearing how you experience your practice in home-based care, what characterises your practice, and how it differs from that of colleagues in a different nursing role. The question did not deliberately explicitly mention “scope of practice,” as nurses in clinical practice rarely use formal SoP terminology; instead, it was intended to capture participants’ responsibilities, activities, and decision-making inherent to their positions in everyday language. Probing prompts such as “Could you elaborate?” or “Can you give an example?” encouraged detailed and reflective responses. The final wording of the open-ended question was informed by insights gained from the preceding structured observations and was piloted for clarity, requiring only minor refinement.

### Data analysis

The Framework Method, adapted for non-dyadic data [24], guided the inductive analysis. This analytic strategy enabled comparison of perspectives that may be less accessible in joint interviews. Separate analytic pathways were intentionally maintained for RNs and non-RNs throughout the early stages of analysis, with convergence of the datasets occurring only from Stage 5 onwards. This approach facilitated analysis at multiple levels: individual interviews, within professional groups, and across professional groups. It enabled identification of overlaps (i.e. similar descriptions reflecting shared concepts and largely congruent conceptualisations) as well as contrasts (i.e. similar descriptions underpinned by different conceptualisations or understandings) in participants’ experiences and interpretations. This analytic strategy provided a more comprehensive account than analysis of individual interviews alone, thereby supporting a more comprehensive account of participants’ perspectives [24, 38].

The analysis was conducted collaboratively by the research team to enhance rigour and reflexivity and comprised eight iterative stages. Stage 1 involved transcription and de-identification of interviews, followed by Stage 2, familiarisation through repeated reading of transcripts. In Stage 3, relevant content (text excerpts) was extracted and coded in relation to the study focus. Inter-rater reliability was assessed by randomly selecting one nurse dyad, with all team members independently extracting content corresponding to the interview question from the first four transcript pages of each dyad participant; the agreement rate was 98%. In Stage 4, codes were organised into a general thematic table (Table 1). Data were coded using a primarily inductive approach, allowing the general themes to emerge directly from participants’ accounts while remaining informed by the study’s conceptual focus on scope of practice. Stage 5 initiated dyadic analysis, during which dyadic codes were developed by bringing together codes from each individual interview within dyads and between dyads. Stage 6 involved development of an analytical framework, Stage 7 its iterative refinement, and Stage 8 application of the framework, together with insights from earlier stages, to guide interpretation. The process was highly iterative, with theme development and refinement continuing as familiarity with the data deepened (Table 1).

**Table 1:** Overview of the framework analysis process

**General theme II: The contours of the nurses’ responsibilities (role)**						
**DYAD 7**	**RN**		**Non-RN**			
	Transcript quote 21	Coding	Transcript quote 3	Coding	Characteristics	
	*“Or*,* for example*,* if there’s a user who has become a bit forgetful*,* then it can just… suddenly happen. Something just arises out of the blue. It might be a small hint that something new is starting to occur with that user. Or*,* for example*,* when we empty urine and so on — if there’s a bad smell*,* or if the user complains about something*,* that’s usually how we notice things. Most of what we pick up comes from what the users tell us. But also*,* when we assist with personal care*,* like morning care*,* we observe their back and notice if anything looks unusual*,* and then we start taking appropriate measures.”*	Vital part of RN activities is observing, assessing, ordinate actions and evaluate – descriptions reflect how she constantly notices (observe and assess) while doing home visits and performing activities	“*Because you also get the full picture of their health that way. For example*,* if you visit someone who normally… yes*,* who’s usually happy to chat*,* and then suddenly they become a bit short or abrupt. If you ask*,* “Are you having a bad day today?” you can quickly see if something is wrong. You wouldn’t notice that if you didn’t know them well.”*	Observing and assessing based on professional competence and experience of the user	**Ongoing clinical observations**	
**General theme III: The contours of the nurses’ activities and tasks (function)**						
**DYAD 1**	**RN**		** Non-RN**			
	Transcript quote 3	Coding	Transcript quote 4	Coding	Characteristics	
	*“No*,* it’s quite a variety of things. It ranges from preparing food and drinks*,* to personal care. It can even include tidying up. Washing up is part of the tasks as well. Then there’s*,* for example*,* wound care and medication*,* where we add something to the dosette. It can also involve refilling Evondos*,* which is a kind of medication dispenser if we have it set up that way — multidose. It can include nursing procedures*,* catheterisation*,* or care of a PICC line. And then there are some things that are more like supervision or monitoring. For instance*,* if someone wants us to check on their mental well-being*,* that’s something we do too. So*,* really*,* the tasks are very*,* very varied. Most of them are the planned ones*,* but sometimes you must go in and make an assessment — take measurements and decide whether you need to call the out-of-hours doctor or if it can wait until the GP.”*	Activities cover basic care household chores to medicine and medico technical tasks as well as administration Oversight and assessments when to step up	“*So*,* it can be anything from putting on compression stockings*,* preparing food*,* giving medication*,* to personal care. Yes*,* yes — food*,* medication*,* and personal care. That’s mainly what those of us who aren’t nurses do. But occasionally*,* there is a bit of wound care as well. […] Yes*,* quite a few of the healthcare professionals handle that (wound care). So*,* once you’ve finished with the first person*,* you move on to the next*,* and that’s how the day continues.”*	Days filled with mainly basic care and some simpler medico technical procedures	**From household chores to advanced care**	
**General theme IV: Parallel scope, divergent responsibilities**						
**DYAD 2**	** RN**		** Non-RN**			
	Transcript quote 10	Coding	Transcript quote 3	Coding	Characteristics	
	*“Yes*,* but as a RN*,* I am usually responsible for those who are in the poorest condition… Yes*,* we often have the medical responsibility. I would say we are responsible for wounds*,* wound care*,* observing whether wounds are being treated properly*,* whether the care is sufficient or done correctly*,* or if a procedure needs to be changed — for example*,* setting up new procedures*,* we are often responsible for that. We also often have responsibility for medications. […] We are responsible when someone comes home from hospital. […] Yes*,* in a way*,* we have responsibility for those with medication management and wound care*,* I would say.*	RN always responsible for the users with most care needs, transitions homecare – hospital and the advanced medico-technical tasks	*“… but they have more expertise than us. Always. We call them*,* or… sometimes we don’t make the assessment ourselves*,* but we collaborate. We collaborate also because the nurses have more competence. … No*,* they’ve been to school longer than us. They are more familiar with diseases and anatomy.”*	Acknowledging two professionally different competences and more in-depth knowledge	**Same, same but different**	

Abbreviations: RN = Registered Nurse, Non-RN = Non-Registered Nurse

### Ethical considerations

This study was conducted in line with the established ethical guidelines of the Declaration of Helsinki [39], with general principles focused on the participants’ health and interests, and acting as a guarantor for the respondents’ autonomy and integrity. It was reviewed by relevant ethical bodies in both Norway and Sweden: the Regional Committees for Medical and Health Research Ethics in Norway (ID 733476), the Norwegian Agency for Shared Services in Education and Research (ID 349256), the Research Ethics Committee at Karlstad University, Sweden (ID C2024/636), and the Swedish Ethical Review Authority (Dnr 2025-00816-01). The Regional Committees in Norway and the Swedish Ethical Review Authority raised no ethical objections but concluded that the project did not fall within their remit. All participants received verbal and written information about the study and provided written informed consent.

### Rigour and reflexivity

All authors of this study are RNs from Norway or Sweden. The research team comprised two professors of nursing, one reader of nursing, one doctoral-level nurse researcher, and one PhD student in nursing science. Three members had extensive experience in long-term care, including home-based care, while two had limited prior experience in this setting. The team’s combined research expertise and diversity of clinical backgrounds supported ongoing discussions and reflexive consideration throughout the study.

The first author conducted all interviews and had previously observed the participants in their work; this prior engagement was actively considered during data collection, analysis, and manuscript preparation. Regular team meetings facilitated reflection on naive readings, interpretations, and potential preconceptions.

## Results

Eight nurse dyads were included in this study. The RNs comprised five females and three males, with a mean age of 40.1 years (range = 24–68; SD = 15). The non-RNs included five females and three males, with a mean age of 49.4 years (range = 40–56; SD = 5.8). Overall, the mean age of the sixteen participants was 44.8 years (range = 24–68; SD = 12). Work experience ranged from 10 months to 29 years, with RNs having a mean of 10.7 years’ experience and non-RNs a mean of 17 years (overall mean = 13.8). Six participants had completed further education, two of whom were non-RNs (Table 2).

The framework analysis identified four overarching themes: modulators of the professional practice environment; the contours of nurses’ responsibilities (role); the contours of nurses’ activities and tasks (function); and parallel scope, divergent responsibilities. Together, these themes reflected both overlapping and contrasting descriptions of how nurses characterised their own practice and that of their colleagues in a different nursing role.

**Table 2:** Participant characteristics

Dyad numbers	Age (years)	Working experience (years)
Dyad I: RN	25–30	3
Dyad I: Non-RN	50–55	26
Dyad II: RN	50–55	25
Dyad II: Non-RN	40–45	12
Dyad III: RN	65–70	19
Dyad III: Non-RN	50–55	15
Dyad IV: RN	40–45	10
Dyad IV: Non-RN	40–45	19
Dyad V: RN	40–45	19
Dyad V: Non-RN	50–55	4
Dyad VI: RN	30–35	3.5
Dyad VI: Non-RN	50–55	22
Dyad VII: RN	20–25	1
Dyad VII: Non-RN	55–60	29
Dyad VIII: RN	30–35	5
Dyad VIII: Non-RN	45–50	9

Abbreviations: RN= Registred Nurse, Non-RN = Non-Registed Nurse

### General Theme 1: Modulators of the professional practice environment

The first general theme, modulators of the professional practice environment, was articulated through four characteristics: an unpredictable environment, a constant re-shuffling of the action list, bugs in the environment, and alone but not lonely. In accordance with the dyadic framework analytic approach, the first three characteristics were largely overlapping across the nurses' accounts, whereas alone but not lonely reflected similar experiential descriptions with differing connotations.

The professional practice environment was described as characterised by uncertainty and continually shifting prerequisites for fulfilling both role and function. This unpredictability was described through accounts of never knowing a care recipient’s condition when opening the door, or what unexpected situations might arise. The nurses described the need to avoid complacency by remaining mentally prepared, ensuring access to potentially required materials and equipment, and staying several steps ahead to respond to unforeseen situations (Table 3).

**Table 3:** An unpredictable environment

RN	Dyad 5	Non-RN
*‘So*,* if there are many admission reports*,* then you check whether there’s anything you need to respond to. And you answer phone calls. People call us – users*,* relatives*,* people from the hospital or the out-of-hours service. It doesn’t happen every day*,* but suddenly it does*,* and it’s the responsible nurse who must answer*,* who has a role in getting things in place and responding to all those questions. … So*,* you always must be two or three steps ahead*.’		‘*We never know what will happen when we open their doors*,* right? A lot of things can happen — they might have fallen*,* and you may need to call an ambulance. Anything can happen*,* so you’re never completely sure of what you’re walking into when you enter that flat*,* you know?’*

The professional practice environment was also described as involving unremitting flexibility and continual re-shuffling of the daily action list. The nurses described reorganising visit sequences, trading visits with colleagues, and adapting care delivery to users’ preferences during visits (Table 4). While this flexibility was described in overlapping ways by both groups, RNs more often described its necessity for managing organisational demands and supporting colleagues in acute situations, whereas non-RNs more frequently linked flexibility to visit order and user preferences.

**Table 4:** A constant re-shuffling of the action list

RN	Dyad 7	Non-RN
*‘It’s just about trying to find a bit of time. If I don’t manage to get all the tasks done in a day*,* then I look at whether it’s because I’m focusing on the essential tasks for that day. If there is something left*,* I prioritise whether I can leave it*,* or whether it’s something I can do another day instead. ’*		*‘So*,* it’s something you think a lot about as you go out and start figuring out how the list should be organised so that it works as well as possible for me too*,* so that I don’t have to drive back and forth. […] So that not so much time is lost on driving. … The driving time isn’t included in the lists; it’s just added on top.’*

Several bugs within the professional practice environment were described in overlapping ways. These included a lack of functional equipment, unsupportive home environments, increasing numbers of visits combined with reduced time for care delivery, and limited opportunities for clinical observation (Table 5). The nurses described these challenges as intensifying in relation to organisational and systemic changes, resulting in stress, concerns about care adequacy, a fast-paced work environment, omissions in care, and suspicions that colleagues were cutting corners. The RNs additionally described challenges related to ensuring appropriate competence among staff, uncertainty regarding sources of support, and difficulties arising when responsibilities “fell between chairs.” These included task-shifting without adequate follow-up, lack of essential equipment, and shortcomings in assessment, documentation, reporting, and follow-up. RNs alone also reported increased responsibility during periods when managers were absent or unavailable.

**Table 5:** Bugs in the environment

RN	Dyad 1	Non-RN
*‘And then*,* as I understand it*,* it’s the department manager’s responsibility to provide us with the right equipment — equipment that works. And that’s where we had another problem*,* which is a bit specific to home nursing: the equipment often can’t tolerate the cold. So*,* if you bring your bag with you and it’s been left out in the car*,* some of the equipment can get damaged. Because it’s often cold out there*,* since we use electric cars*,* and they switch off when we go in to see the users’*		*‘Well*,* there are some people who move very quickly and probably suspect that not everyone is always offered the help they really should be offered. Yes*,* that’s the case. But then there are some for whom it’s more important that they are offered the support they should receive’*

The final characteristic reflected a balance between working independently and having access to collegial support. Although described using similar language, this carried different connotations for the nurses. Both groups described being alone during visits, with some highlighting positive aspects such as time for reflection. However, RNs more often described independent decision-making, whereas non-RNs highlighted the reassurance in knowing they could contact an RN for support when needed (Table 6).

**Table 6:** Alone but not lonely

RN	Dyad 3	Non-RN
*‘But I make most of the decisions myself. If something comes up and there’s another nurse on duty*,* then sometimes I’ve called and asked*,* “What do you think I should do?” Or she or he calls me and asks*,* “What do you think I should do?” But when there aren’t two of us*,* then I rarely call another colleague. There is someone I can call […] I ask him*,* because he knows everyone so well.’*		*‘We are a team*,* but still*,* when we’re out in the field*,* we are on our own. We do have support behind us from other professionals*,* but in those moments when we’re with the service user*,* we are independent. We are alone in there. It’s up to us to carry out our work and observe the users—if something happens or anything changes. Because I can’t just call the nurse and say: “I can see that she’s unwell.’*

### General Theme 2: The contours of the nurses’ responsibilities (role)

The second general theme, the contours of the nurses’ responsibilities (role), was nuanced through two characteristics: ongoing clinical observation and administration versus point-of-care responsibilities. The first characteristic was largely overlapping, while the second reflected similar descriptions with some differing emphases.

The nurses described their responsibilities as encompassing continuous observation to detect deviations from a user’s habitual status, assessment of potential causes, initiation or coordination of appropriate actions, and evaluation of outcomes. They emphasised knowledge of users and continuity of care as central to early detection of change. However, the RNs alone highlighted a tension between improved detection following short gaps between visits and the risk that longer intervals could delay identification of deterioration. They also stressed the need for sustained vigilance, even during basic tasks and under time pressure, particularly in complex situations where changes could occur rapidly and delayed detection could have serious consequences. The non-RNs additionally, and besides their described responsibility for continuous observations, underlined independent reporting, and in some cases decision-making, although with less analytic elaboration (Table 7).

**Table 7:** Ongoing clinical observation

RN	Dyad 2	Non-RN
*‘But as a nurse*,* I do observe everyone. I’m constantly noticing how they are*,* and I know many of them quite well. So*,* from the moment I come in and greet them*,* I notice how awake they are and whether they recognise me… and from the contact I have with them I can tell whether they’re sitting up watching television*,* following the news*,* or lying in bed. Some show very little interest in my presence*,* seem tired*,* or don’t really have the energy to get up or to talk. So*,* the spectrum is quite wide. But these are things I’m continuously observing. And it’s important for us to document this*,* so that we can follow it up properly.’*		*‘We observe*,* for example*,* when we serve food. He eats very little*,* or much less than before. And there are also some patients who usually dress themselves or take care of their personal hygiene*,* and then suddenly one day they don’t – they don’t look properly dressed*,* right? They may not even have their clothes on. So*,* we think that something has changed. It means their abilities may have decreased.’*

The contours of the nurses’ responsibilities were further nuanced through accounts highlighting the interplay between administrative and point-of-care responsibilities. Although described in overlapping ways, this characteristic also reflected some distinctions between the nurses.

Accounts described how nurses’ administrative responsibilities were closely intertwined with the delivery of high-quality point-of-care. Tasks such as updating care plans, ensuring their accuracy and completeness, ordering and managing medications, and preparing necessary equipment and materials were described as shaping the prerequisites for effective bedside care. The nurses described shared responsibilities in ensuring that users received appropriate care and that essential aids were available during visits. They described that these administrative activities were not merely clerical but directly supported safe, timely, and personalised care. While both professional groups stressed the importance of teamwork and mutual support, the RNs alone described a broader scope of administrative and coordination responsibilities. This included overseeing colleagues’ well-being, monitoring workload distribution, providing decision-making support, and following up on completion of care tasks (Table 8). They were also responsible for ensuring adequate staffing levels, maintaining professional competence within the team in the absence of managers, liaising with GPs, hospitals, and other significant stakeholders, and coordinating the users’ overall medical and care trajectory. Additionally, the RNs described taking ownership of creating, updating, and validating care plans, as well as ordering, preparing, and managing medications, reflecting a dual focus on clinical and organisational oversight.

**Table 8:** Admin vs. point-of-care

RN	Dyad 6	Non-RN
*‘My role? Yes – for example*,* I make changes to the care plans for the service users. If a service user has seen the doctor and the doctor has prescribed sleeping tablets for four weeks*,* I need to make sure the care plan is updated. That way*,* the staff who visit the service user can see that they are now on sleeping medication. And if the doctor later stops the medication*,* then I must remove it from the plan*,* so that we are always following up and keeping it accurate. ’*		*‘So*,* if it’s a multi-dose roll*,* and something is missing*,* we just take it from the back of the roll*,* and then we order it for the next delivery from the pharmacy. We order extra*,* or it’s the RNs who do that. I don’t have access to the digital ordering system*,* so I let a RN know. They order an extra daily dose for the next roll.’*

The non-RNs described differences in the distribution of administrative and point-of-care time, noting that the RNs spent more time than them on administrative work. Reactions varied: some expressed frustration, while others described deliberately shifting administrative tasks to the RNs or emphasised the importance of detailed up-to-date care plans for optimal care delivery. The RNs alone stressed that the scope and volume of administrative responsibility were substantial and that insufficient time for these tasks increased the risk of missed or omitted care.

### General Theme 3: The contours of the nurses’ activities and tasks (function)

The third general theme, the contours of the nurses’ activities and tasks (function), was nuanced through two interrelated characteristics: continuity as the bedrock of quality of care, and from household chores to advanced care. While continuity was described in largely overlapping ways by the nurses, the breadth of activities and tasks reflected both overlap and differing connotations in how responsibilities were understood and enacted.

The contours of their activities and tasks were described in overlapping ways, and they described detailed knowledge of users, encompassing preferences, routines, habitual functional status, and typical patterns of behaviour, as fundamental to performing activities effectively and in a tailored manner (Table 9). This continuity of knowledge was described as enabling early detection of change, more rapid and appropriate responses, and increased user comfort, trust, and sense of safety. They emphasised that repeated encounters with the same users supported subtle observational work that could not easily be captured in care plans alone. When continuity was lacking, they described how they adapted their practice by relying more heavily on documentation, care plans, and handovers from colleagues, while simultaneously acknowledging that this could not fully compensate for embodied, experiential knowledge of the user.

**Table 9:** Continuity, the bedrock of quality of care

RN	Dyad 1	Non-RN
*‘Try to follow up with the users you’ve been assigned as much as possible to maintain continuity in the work. And it’s also because they expect that more people with dementia will continue living at home*,* and it’s good for them to have a few regular carers they can relate to—people who know their routines and can gradually adapt to them as well.’*		*‘Things are much easier to understand if you’ve visited the person several times*,* yes. And things are much simpler when you can go to the same person repeatedly and get to know them. It’s easier for us*,* and it’s easier for those who receive our support.’*

Beyond continuity, the nurses described a broad range of activities and tasks extending from household chores to advanced clinical care. In overlapping accounts, they described engaging in activities such as changing bedding, washing dishes, tidying, doing laundry, and addressing minor safety hazards in users’ homes (Table 10). Although these activities were typically outside formal action lists, nurses consistently described them as integral to users’ quality of life and, in some cases, essential to maintaining safety and dignity in the home setting. They also emphasised the need to balance these activities against time constraints and competing clinical demands.

**Table 10:** From household chores to advanced care

RN	Dyad 7	Non-RN
*‘But sometimes*,* if the relatives don’t live nearby*,* we also do the washing up. If we prepare something and there are dishes*,* we wash them*,* and we also help with the laundry.’*		*‘…there might be rugs they could trip over*,* so we straighten them*,* or perhaps ask whether they should be removed. And sometimes a light bulb goes*,* and we must replace it. But mostly we tidy up a bit and do the things that help the place look presentable. Of course*,* there are limits to how much we can tidy up*,* because we don’t have much time for that.’*

In contrast to household tasks, basic care activities, including personal hygiene, dressing, meal preparation, assistance with compression stockings, and support with sleep routines, were described as forming part of the daily work of the nurses. Similarly, simpler medical-technical procedures were described as shared activities, including oral and subcutaneous medication administration, basic wound and stoma care, intermittent catheterisation of females, dialysis-related tasks, blood glucose monitoring, and basic clinical assessment and decision-making.

The RNs were additionally described as undertaking advanced care procedures that extended beyond the scope of the non-RNs. These included intramuscular and intravenous therapy, sterile procedures, medication preparation and management, intermittent catheterisation of both females and males, and complex clinical assessment and decision-making (Table 11). The RNs emphasised that these advanced tasks required not only technical competence but also responsibility for evaluating risk, coordinating care, and responding to rapidly changing clinical situations. Here, the non-RNs, while acknowledging these distinctions, highlighted their own involvement in acute situations, including recognising deterioration, initiating emergency responses, and providing immediate support until further assistance was available.

**Table 11:** From household chores to advanced care

RN	Dyad 4	Non-RN
*‘There are also several service users who need help with meals …In those cases*,* we assist with meals*,* sleep routines*,* personal care*,* and some nursing procedures. Medication management is also included. So*,* there are many different tasks that we carry out… Yes*,* there are several nursing tasks that others cannot carry out. Medication*,* for example – that is our responsibility. Everything related to medicines …is a nursing duty.There are also several procedures that are specifically nursing procedures*,* such as catheter care*,* fluid therapy*,* and everything involving intravenous or sterile procedures.’*		*‘We may have to help them put on their support stockings*,* and sometimes we must call the emergency department. And then there are situations where we must save their life in an acute situation. So*,* the range of what we do is quite wide. There are many different tasks involved.’*

While many descriptions of activities and tasks overlapped, differing connotations were evident. Some RNs highlighted the value of non-RNs performing certain medical-technical procedures as alleviating workload and reducing pressure on RNs. Conversely, some non-RNs expressed frustration with systemic changes that restricted advanced procedures to RNs, particularly when they felt excluded from caring for seriously ill users despite having extensive knowledge of the individual. These accounts illustrate how functional overlap coexisted with boundaries that were experienced as both necessary and, at times, contested.

### General Theme 4: Parallel scope, divergent responsibilities

The fourth general theme, parallel scope, divergent responsibilities, was nuanced through the characteristic: Same, same but different. This characteristic reflected both overlap and similar descriptions with some distinguishing connotations.

Nurses’ parallel scope but divergent responsibilities was depicted as involving largely the same activities and tasks, albeit with distinct areas of responsibility. Although accounts were overlapping, some differences were evident. The nurses agreed that they performed much the same activities, and that RN-specific tasks could at times be difficult to distinguish, noting that the RNs also assisted with personal hygiene and meal preparation. However, the non-RNs expressed frustration that some RNs were reluctant to engage in the most basic activities, such as personal hygiene. Despite these mostly overlapping accounts of nurses’ activities, certain responsibilities differed. The RNs were primarily responsible when users were seriously ill, when holistic assessment was required, during transitions from hospital to home, or in acute situations. At the same time, the nurses stressed the substantial responsibilities held by non-RNs in home-based care. The RNs additionally highlighted the value of competent non-RNs in supporting care delivery and reducing the risk of errors by relieving pressure on them (Table 12).

**Table 12:** Same, same but different

RN	Dyad 5	Non-RN
*‘A doctor cannot function without a RN. It’s exactly the same here. An RN without a skilled non-RN is not quite one hundred per cent a RN. You need a good non-RN around you — someone who understands their role*,* supports you*,* and helps you. […] If everything falls on you*,* there is a high risk of making mistakes.’*		*‘If that’s what you mean*,* then of course the nurse always makes the final assessment. That is their [RNs] responsibility*,* isn’t it? But we [non-RNs] also assess — in fact*,* we have to assess. It’s not that we can; we must.’*

The RNs were described as responsible for medication management, medical care, communication with GPs, development of specific procedures, and advanced clinical decision-making. Some of these responsibilities, such as communication with GPs, were reported to shift temporarily to non-RNs in the absence of a responsible RN. The non-RNs were also described as “first in line” observing changes and reporting back to RNs. There was a shared understanding of differences in knowledge and competence between the two roles: most non-RNs described recognising when RNs’ expertise was required, while some RNs reported being expected, or expecting themselves, to detect more subtle changes than non-RNs. RNs also described having responsibility for supporting non-RNs in communication with users when advanced knowledge was needed to explain procedures or treatments. However, some accounts reflected dissatisfaction or disagreement with the distribution of responsibility. Some non-RNs described not prioritising contact with RNs in acute situations, instead making an autonomous decision to contact a doctor or emergency services. There was also disagreement regarding the need for the RNs to always hold responsibility for users who were seriously ill and during transitions from hospital to home (Table 13).

**Table 13:** Same, same but different

RN	Dyad 3	Non-RN
*‘I think it’s more in relation to if something acute happens*,* or if they have questions about medications [what distinguishes RN from non-RN]. Also*,* perhaps sometimes not everyone — then it becomes an overall assessment.’*		*‘Because I’ve also seen it with very seriously ill patients. It’s not only RNs who can go there; it’s not necessary when the patient is unstable or critically ill. There are those who are very unwell but stable*,* right? And in those cases*,* you don’t need 100% RN care. Why couldn’t a non-RN go there? On the days when there isn’t a RN available*,* what happens then? We haven’t been there*,* and we don’t know them [says RN].’*

## Discussion

In this paper, we explored how nurses experienced their own and each other’s SoP, and what characterised their SoP in home-based care. Using framework analysis, we identified four overarching themes: modulators of the professional practice environment; the contours of nurses’ responsibilities (role); the contours of nurses’ activities and tasks (function); and parallel scope, divergent responsibilities. Each theme was further nuanced through characteristics reflecting the nurses’ descriptions and experiences. The discussion that follows focuses on the most salient findings and considers how nurses’ SoP in home-based care is shaped by the contextual conditions in which nursing is enacted.

Within these contextual conditions, the demanding and unpredictable nature of home-based care requires nurses to combine professional expertise with personal adaptability to respond effectively to complex modulators of practice. Consistent with previous research [40], nurses experienced their professional practice environment as fundamentally unpredictable and increasingly shaped by cumulative pressures, including limited organisational and collegial support, persistent time constraints, and challenges related to professional competence. These modulators were described as affecting both nurses and their service users, contributing to experiences of stress, mental strain, and accelerated or unfinished care. Nurses were required to remain adaptable, both in direct interactions with service users and in care coordination. These demands were further intensified by the structural reality that most home visits are conducted by a single nurse, which heightens the importance of professional independence and a clear understanding of one’s competence and autonomy. Contemporary perspectives increasingly characterise nursing as a complex intervention rather than a set of discrete tasks, embedded within a complex system of interacting components [9, 41]. From this systems viewpoint, phenomena such as scope of practice (SoP) can be understood as system-level events within the professional practice environment, shaped dynamically by organisational structures, practitioner actions, and contextual modulators, rather than as isolated behaviours. Empirical evidence shows that SoP is influenced by factors such as workload, interruptions, staffing constraints, and organisational pressures, which directly affect responsibilities, tasks, and decision-making in both home- and facility-based care [4, 5].

Despite well-documented negative effects of stress, mental strain, and unfinished care on nurses’ retention and ability to fulfil their roles [42, 43], there appears to be limited evidence of sustained change in practice. This lack of action may reinforce negative perceptions of home-based care and may further undermine retention of competent nurses [44]. Our findings indicate that nursing phenomena like SoP operate as complex, system-level events influenced by numerous stakeholders and contextual factors rather than as a set of discrete tasks. The Medical Research Council's Frameworks for complex interventions further emphasise that context and its modulators interact with roles, norms, and care processes [41], underlining that understanding SoP in isolation risks oversimplification. In response to these challenging conditions, nurses must condense the nursing process into brief, time-limited encounters, necessitating continuous assessment and prioritisation of care. The nurses described observing, assessing, initiating, and evaluating care, core elements of the nursing process, as ongoing responsibilities embedded across all home visits and activities. This continuous responsibility was intensified by limited access to service users’ daily routines, requiring nurses to maximise insight within short encounters while managing time pressure and restricted opportunities for dialogue. This condensation appeared to be facilitated through an embodied and largely automatic enactment of the nursing process, resembling what Gladwell has described as rapid, experience-based cognition [45]. While this process falls within heuristic decision-making, it extends beyond tacit knowledge [46] or intuition alone [47]. Rather, it reflects a rapid, experience-based process of situational data gathering and interpretation, grounded in pattern recognition. Our findings align with Gladwell’s [45] notion of “thinking without thinking” and with Benner and Tanner’s [48] conceptualisation of intuition.

The literature indicates that nurses in home- and facility-based care tend to draw on experiential and intuitive knowledge, while the reasoning underpinning their decisions often remains implicit [12]. The embodied enactment identified in the current study was evident among the nurses; however, differences emerged in the extent to which each group articulated analytic reasoning, likely reflecting differences in education and professional preparation. Familiarity with service users’ habitual states stood out as essential across professional groups, enabling detection of subtle deviations. RNs especially emphasised the complexity of balancing continuity of care with the need to preserve sufficient cognitive capacity to interpret change. Previous research has highlighted the importance of familiarity [12] and trust [49] in home-based care; our findings extend this knowledge by underscoring the temporal gap between visits as critical for efficient change detection. Furthermore, research has shown that both feeling competent and being recognised for such competence influence nurses’ well-being [50].

Given the expanding scope and growing complexity of home-based care, there is a need to recognise and support the competence required to balance rapid, experience-based decision-making with slower, analytic reasoning. Building on these cognitive and clinical demands, the findings highlight the complexity of RNs’ roles, which encompass multi-layered responsibilities ranging from direct care provision to care coordination and support of nursing staff competence. These responsibilities require broad and adaptable professional capabilities. The nurses engaged in direct and indirect care; however, the RNs in our study held overarching and system-level responsibilities, underscoring their clinical leadership role. Non-RNs primarily assumed direct care responsibilities, including observation and reporting, positioning them as a valuable extension of RN practice while remaining dependent on clear professional boundaries [cf. 9]. The expanding responsibilities of RNs, extending from direct care to staffing, collegial support, and oversight of decision-making, add a system-level dimension to nursing practice and substantially extend expected clinical leadership competence. In contrast to findings from other contexts [4], RNs in this study remained actively engaged in direct care despite increasing indirect and coordinating demands. Studies examining RN-specific teams in home-based care [51], similar to Swedish organisational models, suggest that while predictability and utilisation of RN expertise may improve, responsibility burdens increase and care may become more fragmented. In addition, local managerial influence emerged as a critical modulator of how the nurses' SoP was enacted. Managers structured work allocation, delegated responsibilities, and provided oversight that clearly shaped nurses’ ability to enact their legal and professional remit. In some cases, managerial prioritisation of tasks over professional judgement created tensions between formal responsibility and actual practice. Our findings highlight that organisational leadership not only supports or constrains practice, but managerial practices appear to mediate how both direct and indirect responsibilities are distributed in daily practice. This may expand or constrain both groups’ autonomy, as well as RNs’ clinical decision-making, thereby influencing the enactment of SoP in daily practice.

There is a need to clarify and actively advocate for the professional remit of RNs, particularly in relation to role boundaries, responsibility distribution, and decision-making authority. Without such clarification, there is a risk of role confusion [6] and uncontrolled expansion of RNs' SoP, potentially diluting or erasing the essence of nursing. The latter was reflected in our study as encompassing the integration of professional competence, relational continuity, and accountability for decision-making. While RNs assumed system-level responsibilities and non-RNs provided essential direct care, the nurses contributed to the core nursing function of delivering safe, responsive, and coordinated care. By framing essence in terms of enacted practice rather than abstract traits, we highlight how professional values and competencies can be realised in context. Norwegian legislation [29, 30], formally assigns responsibility for clinical decision-making and delegation to RNs, while non-RNs practise under supervision. However, our participants’ accounts suggest that these are negotiated in practice in response to workload, contextual demands, and organisational expectations. These legal and organisational structures provide a formal framework for SoP, yet their translation into practice appears to be socially mediated and dependent on managerial and team-level regulation.

The breadth of tasks undertaken by the nurses further intensifies challenges related to continuity, directly shaping nurses’ capacity to deliver safe and coherent care. They performed activities ranging from basic domestic tasks to advanced nursing procedures. While this aligns with previous research [5, 52, Flyum et al., manuscript under review], our findings demonstrate that nurses frequently exceed predefined task lists, even when basic activities were formally excluded. This willingness to provide comprehensive care, despite multiple contextual constraints, reflects a strong commitment to nursing values and ethos [53]. At the same time, they were acutely aware that lack of continuity constrained their ability, and that of colleagues, to deliver comprehensive care. Continuity was unanimously emphasised as a fundamental function of nursing in home-based care. In line with research on older people’s experiences [54], our findings highlight the importance of a core group of nurses to foster trust and familiarity, reflecting both longitudinal and relational continuity [55, 56]. Importantly, this study adds a nursing perspective by demonstrating how longitudinal continuity underpins informational continuity, which in turn enables flexible decision-making and tailored care for older people with complex needs.

Despite a shared conceptual understanding of roles and functions, their practical enactment revealed ambiguities that fundamentally challenged professional clarity. A key tension emerged where the nurses engaged in similar activity repertoires while assuming markedly unequal levels of responsibility and attributing different meanings to these differences. This dissonance was particularly evident in relation to responsibility distribution, time allocated for indirect care, and judgements about when RN expertise was required. Such divergences may reflect unclear role boundaries and insufficient communication regarding differentiated responsibilities. These findings highlight potential risks of missed observations, inadequate reporting, missed care, or failure to rescue when care is delivered without appropriate competence. These risks are further amplified by ongoing task-shifting practices, while responsibility shifting often remains implicit and unexamined. Returning to Malone and Cliffe’s [1] conceptualisation of SoP, responsibilities, activities, and tasks must be appropriate to the nurse’s education, competence, and authorisation. Our findings illustrate the tension between the formally regulated scope of practice and how scope is enacted in everyday home-based care. When this principle is overlooked or poorly communicated, risks arise for both nurses and service users, particularly in contexts where responsibility is diffuse and implicit. The dissonance identified underscores the importance of managerial oversight and organisational structures in supporting appropriate enactment of SoP, reinforcing professional boundaries, and enabling safe delegation and leadership.

Our study suggests that the essence of nursing in home-based care extends beyond tasks or legal mandates, residing instead in the integration of clinical judgment, relational continuity, rapid analytic decision-making, and accountability within complex, variable contexts. This understanding, grounded in both legislation and described practice, also reveals how the often ‘invisible’ nature of nursing work can shift responsibilities and create uncertainty about professional scope, underscoring the need for clearer role definition and communication.

### Methodological considerations

The use of a qualitative descriptive design enabled the study to capture participants’ own understanding and descriptions of nursing phenomena, rather than producing highly interpreted results [22]. This design was chosen to ensure the findings remained grounded in participants’ experiences. To enhance transferability, an information-rich sample of RNs and non-RNs working in Norwegian home-based care was recruited, which may be transferable to similar community-based, primary, or long-term care settings, while recognising that transferability is bounded by the organisation of Norwegian home-based care and the broader Nordic welfare model with comparable interprofessional configurations [55]. Sample size was guided by the concept of information power, recognising that smaller, purposive samples can yield sufficient data when the study aim is focused and participants are relatively homogeneous [35, 36].

The prior structured observations in Step I of the project informed the development of the open-ended interview question and probes, as well as the *a posteriori* construction of dyads. This provided contextual knowledge while supporting analytic independence through collaborative team coding and reflexive discussion. These observations form part of the broader DECIDE project, within which the present study represents a second step focusing on interview-based exploration, complementing prior observational work. The findings should therefore be interpreted within this broader study context. Including both RNs and non-RNs in dyads enabled systematic comparison of multiple perspectives within shared contextual conditions. Transferability remains bounded by the organisation of Norwegian home-based care and the broader Nordic welfare model, which may limit applicability to other healthcare systems.

Focused interviews using a single open-ended question with probing prompts enabled participants to reflect openly on their experiences while keeping the interviews aligned with the study aim [22]. This approach facilitated the collection of rich, grounded data that would be difficult to achieve with more standardised methods. All interviews were conducted by the first author to enhance consistency, with review of the first two interviews by senior researchers to support trustworthiness and dependability. Prior observations informed the interview focus, and although potential influence on coding and interpretation was addressed through collaborative analysis and reflexive discussions, such influence cannot be fully excluded and should be considered when interpreting the findings. The interview question was deliberately phrased to capture nurses’ descriptions of their practice in everyday language rather than explicitly asking about scope of practice, reflecting how clinicians naturally discuss their work. Scope of practice was operationalised as a sensitising concept, guiding coding without imposing a formal theoretical framework [19, 20].

Individual interviews (non-dyadic data) were analysed as posterior dyads using the adapted framework method [24]. This analytic strategy facilitated identification of overlaps and contrasts within dyads, within professional groups (RN/non-RN), and between groups, supporting a more comprehensive understanding of similarities and differences across perspectives than analysis of individual interviews alone. Furthermore, coding and analysis were conducted collaboratively by the research team, with interpretive oversight by the last author to ensure analytical coherence and consistency. This approach enabled exploration of multiple levels of understanding within the data and strengthened interpretive depth and analytic robustness in accordance with established trustworthiness criteria in qualitative research [57–59]. The systematic and transparent nature of the framework method [24], combined with team collaboration, consistency checks across researchers, and detailed documentation of analytic decisions, further strengthened the credibility, dependability, and confirmability of the study [57–60]. Nevertheless, analytic decisions remain interpretive and shaped by the study design; as the study is based on participants’ accounts of their practice, the findings reflect experiences and perceptions rather than direct observation of behaviour, and these considerations should be borne in mind when evaluating the findings [60].

## Conclusions and implications

This study suggests that nurses’ SoP in home-based care is shaped by complex contextual modulators, requiring continual professional adaptability in unpredictable and time-constrained environments. Nurses’ responsibilities were characterised by a condensed and embodied enactment of the nursing process, albeit with differentiated levels of responsibilities between the nurses, reflecting rapid, experience-based cognition with more deliberate assessment and prioritisation. Although the nurses articulated a shared conceptual understanding of roles and functions, their practical enactment revealed parallel and overlapping activities alongside divergent responsibilities. The RNs’ SoP was found to be simultaneously expanding and intensifying, encompassing direct care in addition to coordination, clinical leadership, and accountability for care completion. The coexistence of parallel activity repertoires with unequal responsibilities highlights ambiguities in SoP that may contribute to risks of implicit responsibility shifting and missed care. These findings highlight the need for empirically grounded and clinically nuanced descriptions of nurses’ SoP in home-based care that explicitly account for contextual conditions, continuity requirements, and differentiated responsibilities. Such clarity is essential to inform nursing education, support clinical and organisational nursing leadership, strengthen nursing unit culture, and guide future research, and may be important to prioritise before further organisational changes are introduced.

## Data Availability

The raw data that supports the findings of this study are not available due to confidentiality and ethical restrictions including data would not be shared. However, requests for data access can be directed to the institutional Data Protection Officer at mail to: personvernombud@ldh.no, who will evaluate inquiries on a case-by-case basis. The interview guide is available upon reasonable request from the corresponding author.

## Abbreviations

RN: Registered nurse
Non-RN: Non-registered nurse
SoP: Scope of practice

## References

[1] Malone G, Cliffe C. Framework for remote and isolated practice. Canberra: CRANAplus; 2018. Available from: https://s3.ap-southeast-2.amazonaws.com/cranapluswebsite-assets/files/CRANAFramework-for-Remote-and-Isolated-Practice-2018.pdf

[2] Royal College of Nursing. RCN new definition of nursing. London: RCN. 2023. Available from: https://www.rcn.org.uk/-/media/Royal-College-Of-Nursing/Documents/Publications/2023/December/011-253.pdf

[3] International Council of Nurses. Renewing the definitions of nursing and a nurse: final project report. Geneva: ICN; 2025. Available from: https://www.icn.ch/sites/default/files/2025-06/ICN_Definition-Nursing_Report_EN_Web_0.pdf

[4] Nordaunet OM, Gjevjon ER, Aagaard H, et al. Nursing practice in relation to older people’s fundamentals of care in nursing homes. Int J Nurs Stud Adv. 2025;6:100346. 10.1016/j.ijnsa.2025.100346. 10.1016/j.ijnsa.2025.100346 PMC12148421 40491901

[5] Sandberg K, Eklund AJ, Borglin G, et al. Nurses’ scope of practice and fundamental care in relation to older people: an exploratory home-based study. Int J Nurs Stud Adv. 2026;10(100492). 10.1016/j.ijnsa.2026.100492. 10.1016/j.ijnsa.2026.100492 PMC12905735 41695998

[6] Dada OD, Amankwaa I, Brownie S. Perspectives of community mental health nurses as care coordinators within a multidisciplinary team: a systematic review. J Interprof Care. 2025;39(3):499–509. 10.1080/13561820.2025.2487032. 40314258 10.1080/13561820.2025.2487032

[7] Alhassan A, Sencherey V, Bobuafor C, et al. Practices and challenges of supervision and delegation in nursing in Africa: a scoping review. BMC Nurs. 2025;24:1094. 10.1186/s12912-025-03683-9. 10.1186/s12912-025-03683-9 PMC12369257 40841651

[8] Fulham-McQuillan H, Murphy M, Hilliard C, et al. Determinants of the implementation, integration, and sustainability of new non-professionally affiliated psychosocial support roles: a scoping review. BMC Health Serv Res. 2025;25:1538. 10.1186/s12913-025-12615-x. 41299596 10.1186/s12913-025-12615-x PMC12659520

[9] Richards DA, Borglin G. Shitty nursing: the new normal? Int J Nurs Stud. 2019;91:148–52. 10.1016/j.ijnurstu.2018.12.018. 30831477 10.1016/j.ijnurstu.2018.12.018

[10] Tanner CA. Thinking like a nurse: a research-based model of clinical judgment in nursing. J Nurs Educ. 2006;45(6):204–11. 10.3928/01484834-20060601-04. 16780008 10.3928/01484834-20060601-04

[11] Thompson C, Stapley S. Do educational interventions improve nurses’ clinical decision making and judgement? Int J Nurs Stud. 2011;48(7):881–93. 10.1016/j.ijnurstu.2010.12.005. 21241984 10.1016/j.ijnurstu.2010.12.005

[12] Flyum IR, Gjevjon ER, Eklund AJ, et al. What do we know about nursing practice in relation to functional ability limitations, frailty and models of care among older people in home- and facility-based care: a scoping review. BMC Nurs. 2025;24:406. 10.1186/s12912-025-02948-7. 40211311 10.1186/s12912-025-02948-7 PMC11987274

[13] Schubert M, Glass TR, Clarke SP, Aiken LH, Schaffert-Witvliet B, Sloane DM, et al. Rationing of nursing care and its relationship to patient outcomes. Int J Qual Health Care. 2008;20(4):227–37. 10.1093/intqhc/mzn017. 18436556 10.1093/intqhc/mzn017 PMC2582013

[14] Kalánková D, Stolt M, Scott PA, Papastavrou E, Suhonen R, RANCARE COST Action CA15208. Unmet care needs of older people: a scoping review. Nurs Ethics. 2021;28(2):149–78. 10.1177/0969733020948112. 33000674 10.1177/0969733020948112

[15] Henderson V. The concept of nursing. J Adv Nurs. 1978;3(2):113–30. 10.1111/j.1365-2648.1978.tb00949.x. 246439 10.1111/j.1365-2648.1978.tb00837.x

[16] Goodolf DM, Godfrey N. A think tank in action: building new knowledge about professional identity in nursing. J Prof Nurs. 2021;37(2):493–9. 10.1016/j.profnurs.2021.01.014. 33867110 10.1016/j.profnurs.2020.10.007

[17] Andrew N. Professional identity in nursing: are we there yet? Nurse Educ Today. 2012;32(8):846–9. 10.1016/j.nedt.2012.03.011. 22531469 10.1016/j.nedt.2012.03.014

[18] Allen D. The invisible work of nurses: hospitals, organisation and healthcare. Abingdon: Routledge; 2015.

[19] Blumer H. What is wrong with social theory? Am. Sociol Rev. 1954;19(1):3–10. 10.2307/2088165.

[20] Bowen GA. Grounded theory and sensitizing concepts. Int J Qual Methods. 2006;5(3):12–23. 10.1177/160940690600500304

[21] Helgheim BI, Sandbæk B. Who is doing what in home care services? Int J Environ Res Public Health. 2021;18(19):10504. 10.3390/ijerph181910504. 34639804 10.3390/ijerph181910504 PMC8508197

[22] Sandelowski M. Whatever happened to qualitative description? Res Nurs Health. 2000;23(4):334–40. 10.1002/1098-240X. (200008)23:4<334::AID-NUR9>3.0.CO;2-G. 10940958 10.1002/1098-240x(200008)23:4<334::aid-nur9>3.0.co;2-g

[23] Merton RK. The focused interview. New York: Free; 2008.

[24] Collaço N, Wagland R, Alexis O, Gavin A, Glaser A, Watson EK. Using the framework method for the analysis of qualitative dyadic data in health research. Qual Health Res. 2021;31(8):1555–64. 10.1177/10497323211011599. 33980102 10.1177/10497323211011599 PMC8278550

[25] O’Brien BC, Harris IB, Beckman TJ, Reed DA, Cook DA. Standards for reporting qualitative research (SRQR). Acad Med. 2014;89(9):1245–51. 10.1097/ACM.0000000000000388. 24979285 10.1097/ACM.0000000000000388

[26] Saunes IS, Karanikolos M, Sagan A. Norway: health system review. Oslo: Norwegian Institute of Public Health; 2020. 32863241

[27] Ministry of Education and Research. Forskrift om nasjonal retningslinje for sykepleierutdanning [Regulations on national guidelines for nursing education] (FOR-2025-07-04-1477). Oslo: Lovdata. 2025. Available from: https://lovdata.no/dokument/SF/forskrift/2025-07-04-1477

[28] Ministry of Education and Research. Forskrift om felles rammeplan for helse- og sosialfagutdanninger. [Regulations on a common framework plan for health and social care education] (FOR-2025-07-04-1478). Oslo: Lovdata. 2025. Available from: https://lovdata.no/dokument/LTI/forskrift/2025-07-04-1478

[29] Norwegian Ministry of Health and Care Services. Lov om helsepersonell m.v. (LOV-1999-07-02-64) [Health Personnel Act]. Oslo: Lovdata. 1999. Available from: https://lovdata.no/dokument/NL/lov/1999-07-02-64

[30] Norwegian Ministry of Health and Care Services. Lov om kommunale helse- og omsorgstjenester m.m. (LOV-2011-06-24-30) [Municipal Health and Care Services Act]. Oslo: Lovdata. 2011. Available from: https://lovdata.no/dokument/NL/lov/2011-06-24-30

[31] Norwegian Directorate of Health. Helsepersonelloven med kommentarer: krav til helsepersonells yrkesutøvelse [Health Personnel Act with commentary: professional responsibility and delegation]. Oslo: Helsedirektoratet. 2018 [updated 2026]. Available from: https://www.helsedirektoratet.no/rundskriv/helsepersonelloven-med-kommentarer/krav-til-helsepersonells-yrkesutovelse

[32] Norwegian Board of Health Supervision. Administrative reaksjoner overfor helsepersonell og virksomheter i helse- og omsorgstjenesten m.m. [Administrative reactions against health personnel and health and care services]. Oslo: Statens helsetilsyn. 2025. Available from: https://www.helsetilsynet.no/tilsyn/om-tilsynssaker/reaksjonsformer-personell-virksomheter-i-helse-omsorgstjenesten/

[33] Emmel N. Sampling and choosing cases in qualitative research: a realist approach. London: SAGE; 2013. 10.4135/9781473913882.

[34] Patton MQ. Qualitative research and evaluation methods. 4th ed. Thousand Oaks (CA): SAGE; 2015.

[35] Guest G, Bunce A, Johnson L. How many interviews are enough? An Experiment with Data Saturation and Variability. Field Methods. 2006;18(1):59–82. 10.1177/1525822X05279903.

[36] Malterud K, Siersma VD, Guassora AD. Sample size in qualitative interview studies: guided by information power. Qual Health Res. 2016;26(13):1753–60. 10.1177/1049732315617444. 26613970 10.1177/1049732315617444

[37] Eftekhari H. Transcribing in the digital age: qualitative research practice utilising intelligent speech recognition technology. Eur J Cardiovasc Nurs. 2024. 10.1093/eurjcn/zvae013. 38315187 10.1093/eurjcn/zvae013 PMC11334016

[38] Eisikovits Z, Koren C. Approaches to and outcomes of dyadic interview analysis. Qual Health Res. 2010;20(12):1642–55. 10.1177/1049732310376520. 20663940 10.1177/1049732310376520

[39] World Medical Association. Declaration of Helsinki: ethical principles for medical research involving human subjects. World Medical Association. 2024. Available from: https://www.wma.net/policies-post/wma-declaration-of-helsinki/

[40] Martinsen B, Mortensen AS, Norlyk A. Nordic homecare nursing from the perspective of homecare nurses: a meta-ethnography. Br J Community Nurs. 2018;23(12):597–604. 10.12968/bjcn.2018.23.12.597. 30521386 10.12968/bjcn.2018.23.12.597

[41] Skivington K, Matthews L, Simpson SA, Craig P, Baird J, Blazeby JM, et al. A new framework for developing and evaluating complex interventions. BMJ. 2021;374:n2061. 10.1136/bmj.n2061. 34593508 10.1136/bmj.n2061 PMC8482308

[42] Khatatbeh H, Pakai A, Al-Dwaikat T, Onchonga D, Amer F, Prémusz V, et al. Nurses’ burnout and quality of life: a systematic review. Nurs Open. 2022;9(3):1564–74. 10.1002/nop2.936. 33991408 10.1002/nop2.936 PMC8994939

[43] Stemmer R, Bassi E, Ezra S, et al. Unfinished nursing care and its impact on nurse outcomes. J Adv Nurs. 2022;78:2290–303. 10.1111/jan.15286. 35533090 10.1111/jan.15286

[44] Kiptulon EK, Elmadani M, Szőllősi A, et al. The race to retain nursing workforce in healthcare: an umbrella review. BMC Health Serv Res. 2025;25:1344. 10.1186/s12913-025-13365-6. 41068746 10.1186/s12913-025-13365-6 PMC12513143

[45] Gladwell M. Blink: the power of thinking without thinking. New York: Little, Brown and Company; 2005.

[46] Acquaviva K, Haskell H, Johnson J. Human cognition and the dynamics of failure to rescue: the Lewis Blackman case. J Prof Nurs. 2013;29(2):95–101. 10.1016/j.profnurs.2012.12.001. 23566455 10.1016/j.profnurs.2012.12.009

[47] Orr E, Jack SM, Campbell K, Strohm S. Valuing tacit nursing knowledge during the COVID-19 pandemic. Public Health Nurs. 2023;40(1):178–81. 10.1111/phn.13129. 36062958 10.1111/phn.13129 PMC9537875

[48] Benner P, Tanner C. Clinical judgment: how expert nurses use intuition. Am J Nurs. 1987;87(1):23–31. 3642979

[49] Capitani N, Rasero L, Longobucco Y, Magi CE, Iovino P, Bambi S, et al. Highlanders: a qualitative study to explore the experiences of home care nurses in rural areas in Italy. Infermieristica J. 2024;3(2):81–92. 10.36253/if.2450.

[50] Andersson H, Lindholm M, Pettersson M, et al. Nurses’ competencies in home healthcare: an interview study. BMC Nurs. 2017;16:65. 10.1186/s12912-017-0264-9. 29176934 10.1186/s12912-017-0264-9 PMC5693583

[51] Thorkildsen T, Karlsen C, Barken T. Nurses’ experiences with home nursing care teams – a qualitative study. Sykepleien Forskning. 2025;20:100995. 10.4220/Sykepleienf.2025.100995en.

[52] Brenne BA, Hedlund M, Ingstad K. Exploring home-based care nurses’ mindset for nursing practices: a phenomenological study. BMC Nurs. 2022;21:291. 10.1186/s12912-022-01068-w. 36316738 10.1186/s12912-022-01068-w PMC9623960

[53] International Council of Nurses. The ICN code of ethics for nurses. Geneva: ICN. 2021. Available from: https://www.icn.ch/resources/publications-and-reports/icn-code-ethics-nurses

[54] Djukanovic I, Hellström A, Wolke A, Schildmeijer K. The meaning of continuity of care from the perspective of older people with complex care needs: a scoping review. Geriatr Nurs. 2024;55:354–61. 10.1016/j.gerinurse.2023.12.012. 38171186 10.1016/j.gerinurse.2023.12.016

[55] Freeman GK, Olesen F, Hjortdahl P. Continuity of care: an essential element of modern general practice? Fam Pract. 2003;20(6):623–7. 10.1093/fampra/cmg601. 14701883 10.1093/fampra/cmg601

[56] Haggerty JL, Reid RJ, Freeman GK, Starfield BH, Adair CE, McKendry R. Continuity of care: a multidisciplinary review. BMJ. 2003;327(7425):1219–21. 10.1136/bmj.327.7425.1219. 14630762 10.1136/bmj.327.7425.1219 PMC274066

[57] Lincoln YS, Guba EG. Naturalistic inquiry. Newbury Park (CA): SAGE; 1985.

[58] Morse JM. Determining sample size. Qual Health Res. 2000;10(1):3–5. 10.1177/104973200129118183.

[59] Stahl NA, King JR. Expanding approaches for research: understanding and using trustworthiness in qualitative research. J Dev Educ. 2020;44(1):26–8.

[60] Benner P. Quality of life: a phenomenological perspective on explanation, prediction, and understanding in nursing science. Adv Nurs Sci. 1985;8(1):1–14. 10.1097/00012272-198510000-00004 10.1097/00012272-198510000-00004 3933410

